# All-in-one 3D printed microscopy chamber for multidimensional imaging, the *UniverSlide*

**DOI:** 10.1038/srep42378

**Published:** 2017-02-10

**Authors:** Kevin Alessandri, Laetitia Andrique, Maxime Feyeux, Andreas Bikfalvi, Pierre Nassoy, Gaëlle Recher

**Affiliations:** 1 LP2N, CNRS UMR 5298, IOA, 1 rue François Mitterrand, 33400 Talence, France; 2 Institut d’Optique Graduate School, IOA, 1 rue François Mitterrand, 33400 Talence, France; 3 Université de Bordeaux, Bordeaux, France; 4 LAMC, Inserm U1029, 63 Avenue des Facultés, 33600 Pessac, France; 5 IMN, CNRS UMR 5293, 146 rue Léo Saignat – CS 61292 - 33076 Bordeaux cedex, France.

## Abstract

While live 3D high resolution microscopy techniques are developing rapidly, their use for biological applications is partially hampered by practical difficulties such as the lack of a versatile sample chamber. Here, we propose the design of a multi-usage observation chamber adapted for live 3D bio-imaging. We show the usefulness and practicality of this chamber, which we named the *UniverSlide*, for live imaging of two case examples, namely multicellular systems encapsulated in sub-millimeter hydrogel shells and zebrafish larvae. We also demonstrate its versatility and compatibility with all microscopy devices by using upright or inverted microscope configurations after loading the *UniverSlide* with fixed or living samples. Further, the device is applicable for medium/high throughput screening and automatized multi-position image acquisition, providing a constraint-free but stable and parallelized immobilization of the samples. The frame of the *UniverSlide* is fabricated using a stereolithography 3D printer, has the size of a microscopy slide, is autoclavable and sealed with a removable lid, which makes it suitable for use in a controlled culture environment. We describe in details how to build this chamber and we provide all the files necessary to print the different pieces in the lab.

Visualising morphogenetic processes and deciphering biomechanical cues within organisms or thick living samples has become instrumental in all fields of biology (developmental biology, stem cells and tissue engineering, biophysics…) but requires to image the samples with good spatial and temporal resolution, repetitively, and in a reasonably high throughput format in order to provide accurate and statistically reliable quantification^1^,^2^,^3^,^4^,^5^,^6^,^7^,^8^. A variety of imaging techniques and configurations are now available in the laboratories and facilities equipped with state-of-the-art microscopes (e.g. confocal, multi-photon or spinning-disk microscopes). Since they are often shared within an imaging platform, these costly microscopes cannot be customized for specific applications. To retain the versatility of the imaging instrument, one solution is to build a multi-stage, omni-sample observation chamber that fulfils the following requirements: tissue-culture grade, high sample content, ease to assemble, transportability, individualization of samples for further identification, immobilization of the samples (for accurate image analysis) without mechanical constraint (that would be damageable for the physiological growth and morphogenesis of the samples) and compatibility with all types of microscopes and stages.

Chambers and devices are usually customized in each laboratory, for each application or type of sample. For example, morphogenesis of the zebrafish larva is investigated by embedding the fish in low melting agarose^9^,^10^. Although this approach is easy to implement, it is not suitable for long term imaging because of the mechanical constraints generated by the gel on growing larva and it does not allow to reach good statistics because of the lack of automatization in the mounting protocol^9^. Other strategies based on arrays of wells with a specific design and shape have been developed for processing the above mentioned issues^11^,^12^. One remaining limitation is that this methodology cannot be transferred to other samples nor microscopes in a straightforward manner^13^,^14^,^15^,^16^,^17^,^18^. There are various examples characteristic for the diversity of biological samples. Zebrafish larvae have an elongated and tortuous shape but they are very tolerant in terms of culture conditions. However, other embryos are rather round but more demanding in terms of environment (buffered and sterile medium). In the case of mouse embryos, several strategies have been pursued. The embryologist’s method consists in collecting and imaging them all together for a given experimental condition in the dish either in drops of medium covered with mineral oil^19^, where they can move with stage displacement. This makes it very difficult to monitor individual embryos over long periods of time while parallelising the imaging procedure in order to provide a population’s statistics. Another method consist in aligning embryos in the interspaces of Nylon mesh^20^. The later provides a well-defined array to park and register the embryos and is cost-effective. However, its assembly and use is highly variable and depends on the skills of the experimentalist. Other minor disadvantages are that embryos might move by sliding over the round section of the mesh wires, and that the material of the mesh is not compatible with two-photon excitation. Finally, there are also commercially available devices specifically designed for mouse embryos (e.g. from Dolomite-microfuidics) but they are more expensive, only compatible with inverted microscopes and require specific stage holders.

Another category of samples for which observation chambers need to be designed and optimized are 3D multicellular aggregates. Proposed as *in vitro* tumour models 30 years ago^21^, they are now the subject of renewed interest because of the advances in optical sectioning microscopy^22^, the development of microfluidics strategies to produce them in a controlled fashion^23^,^24^,^25^, and the necessity for pharmaceutical and cosmetic companies to reduce animal testing for toxicity assays^26^. Initially restricted to the field of cancer biology^21^, the 3D multicellular aggregates have now numerous variants, such as embryoid bodies, organoids, neurospheres^27^,^28^,^29^,^30^,^31^. As previously mentioned for mouse embryos, these *in vitro* 3D cultures have an overall rounded shape, and their growth or survival requires the use of defined media and culture conditions. To achieve high throughput screening of drugs with these *in vitro* models, it is important to perform automatized multiposition imaging either by favouring the speed of acquisition at the expense of spatial resolution or conversely.

Here we present an all-in-one device that overcomes the limitations indicated above and may be used for imaging thick samples in 3D over time. We developed an imaging chamber that can be completely built and assembled in the lab. It requires a 3D printer working with biocompatible resin and with a typical axial resolution of about 25 μm, as well as commonly used lab materials (coverslips, agarose and silicone elastomer). The chamber can be used with all types of commercial microscopes (upright and inverted), all types of stages that accommodate regular microscopy slides, and it is suitable for imaging of a wide variety of samples (all sub-millimetric embryos, tissue explants, or cell aggregates). This versatility led us to name the device *UniveSlide*. In this article we describe in detail how to construct and assemble the chamber, and we present two possible applications that illustrate its potentialities in terms of medium/high throughput 3D + time imaging, corresponding to two different 3D samples: alginate capsules^23^,^32^ filled with a mixture of two fluorescent cells lines, respectively labelled with GFP in the membranes and tdTomato in the nuclei and zebrafish fluorescent transgenic larva. These samples are easily placed in the *UniveSlide* device, can be imaged for hours, and generate datasets providing high quality images that permit individual cell tracking.

## Results

### Procedure for building and assembling the chamber

**Figure 1:**
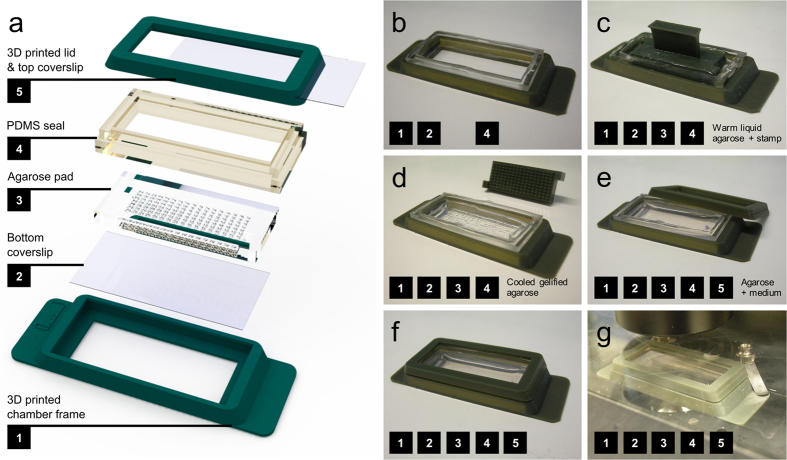
Depiction and assembly of the chamber. (**a**) 3D drawing of the different parts of the chamber, comprising the main chamber frame (1) sized accordingly to a microscopy slide, the bottom coverslip (2) that will allow imaging from inverted microscopes, the agarose pad (3) that is imprinted according to the chosen type of stamp corresponding to the sample of interest (round or elongated for example), the PDMS seal (4) that ensures the ceiling of the chamber and prevents any contact between the content of the chamber and the printed resin and finally the frame of the lid (5) in which the coverslip is slid to allow imaging from top for upright microscopes configuration. (**b**) Assembly of the main chamber frame (1) with the coverslip and the PDMS seal. The most important step here is to clip properly the PDMS in the chamber to ensure no further leakage of the agarose or the medium. (**c**) After the adjunction of 2 mL of warm LMP (Low Melting Point) agarose the stamp is disposed to prevent the formation of bubbles. (**d**) After a cooling step (better is to keep the device at 4 °C for a few minutes), the stamp is removed carefully. Ideally the agarose should be maintained with a thick and smooth device such as for example insect forceps, and if necessary, few drops of medium can be disposed to facilitate the detachment of the agarose from the stamp. (**e**) After removal of the stamp the chamber can be filled with the appropriate medium. If necessary at this step, or with the lid on top (**f**) of the assembly procedure, the device can be placed in the incubator to allow the medium to diffuse in the agarose and to equilibrate in gas. After having loaded the samples within the holes, the chamber can be placed on the stage of the microscope (**g**).

In the last decade the technical specifications and the variety of table-top 3D printers have drastically evolved, which contributed to make them inexpensive and widespread as standard lab equipment. They serve to design and build customized tools for scientists. We took advantage of this technology to design an imaging chamber that is easy-to-build and easy-to-use in optical microscopy applications. We used the Micro Plus Hi-Re (Envisiontech, CITY) 3D-printer with the biocompatible HTM140 resin (Envisiontech, CITY). Although not directly tested, we however anticipate that any standard resin could also be used since glass and crosslinked polydimethylsiloxane (PDMS) are the only two materials in contact with the biological sample. First, to obtain compatibility with the majority of microscope stages, the size of the *UniverSlide* has been chosen to be the one of a regular microscopy slide, i.e. 26 × 76 mm^2^. The whole device then comprises five main parts which are designated in Fig. 1a. The basement of the chamber is widely opened to provide the largest field of view and is the receptacle for the borosilicate coverslip (24 × 50 mm^2^). The glass slide is placed at the bottom of the frame (Fig. 1a and Supplementary Fig. S1) No glue is used for sealing in order to avoid any risk of cellular toxicity. Instead, we made a rectangular flexible PDMS seal, which is prepared by using a printed resin frame as a mould (Fig. 1b). The whole assembly procedure is described in the legend of Supplementary Fig. S2 (Fig. S2). The PDMS seal is clipped in the tank and sticks to the bottom cover-glass. Then, agarose, whose concentration and grade are chosen depending on sample requirements, is poured into the tank (~2 mL) and the appropriate stamp (chosen among the different models available, see Supplementary Fig. S3) is apposed with care to prevent the formation of bubbles (Fig. 1c). Removal of the stamp (Fig. 1d) can be facilitated by the addition of medium. The tank is then filled with the medium of choice (Fig. 1e) and samples are loaded in the wells (see next section for details). After placing the lid on (Fig. 1f, Supplementary Fig. S1), the chamber is ready for microscopy use (Fig. 1g).

The overall time for *UniverSlide* assembly is about 10 minutes for an untrained experimentalist. Loading the well with cellular capsules or larvae can be performed manually in a very effective manner, as described in the following section.

### Optimized design of the wells for capsules mounting

We applied standard mouse embryos manipulation protocols using homemade pulled pipettes and mouth aspiration to load encapsulated cellular aggregates into the *UniverSlide*^19^. Note that gel-loading-tips’ associated with high-precision automatic pipettor are a possible alternative although slightly less effective in our hands.

**Figure 2:**
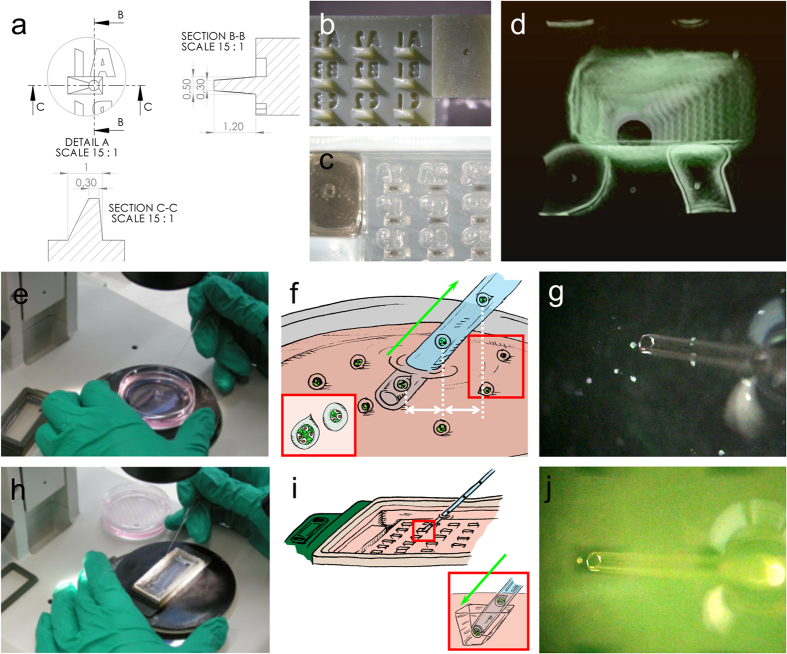
The shape of the wells facilitates loading of the chamber with cells enclosing alginate capsules. (**a**) Technical drawing of the well with sizes. (**b**) Close-up of the 3D-printed stamp wells imprints are protruding whereas letters are hollowed. (**c**) Solidified agarose gel pad after imprinting; bumps and cavities are now inverted. Note that a part of the resin block is used to hold the stamp in place [visible on the top right of (**b**)] and to imprint a cuvette in the upper left corner. This cuvette is used for medium exchange since a tip can be inserted here without any risk of touching the samples. (**d**) 3D rendering of the agarose wells after fluorescence labelling of the agarose with Dextran (see Movie 1). (**e**–**j**) Capsules manipulation is achieved by visualization with a stereomicroscope for both loading the capsules in the pipette (**e**–**g**) and placing them in the chamber (**h**–**j**). (**f** and **i**) Drawings illustrating the procedure for collecting the capsules. Capsules residing in a 60 mm petri dish in warmed medium are carefully and regularly aspirated into the mouth pipette, ensuring regular spacing (**h**). They are then released in each well to fill the whole frame of the chamber (**i**). The inset illustrates the shape of the well that has been designed to permit the insertion of the pipette (67.5° slope on the right side) and to minimize the optical aberrations (80° slope on the right side) when looking from top [for detailed dimensions, see (**a**)].

All the 3D-printed parts were drawn using SolidWorks. Special care was taken to design the stamp structures that will give rise to agarose wells (Fig. 2a). Although the pattern is drawn to better fit with sample shape, some common crucial features are conserved. First, one side of the well is designed with a gentle slope (taken to be 67.5° with respect to the vertical axis) so that the pipette can be inserted without touching and damaging the agarose matrix (Fig. 2i, inset). Second, the bottom of the well is a flat circular surface (diameter, 300 μm). The capsules are trapped in the wells given the height of the well (1200 μm >> capsule diameter ~200 μm) but they are not mechanically constrained as they are in all classical embedding approaches. Third, a given microstructured tip is replicated tens of times on the moulding stamp surface to generate an array (Fig. 2b) of tips that will form the wells upon imprinting into the agarose pad (Fig. 2c). For instance, the moulding stamp used in most of the experiments described in this work was designed to generate 153 wells (17 columns and 9 rows), which is the highest number of wells that the useable surface of the coverslip can accommodate without any overlap. Fourth, a label composed of a character and a number (respectively for line and column) is assigned to each micro-structured tip, which allows practical registration for low magnification imaging. A 3D rendering of the shape of the well shows how accurately the tip printed on the stamp transferred to the agarose pad (Fig. 2d and Movie 1).

Filling the wells with the samples (cellular capsules in Fig. 2Ee–g) starts by mouth pipetting the capsules initially placed in a petri dish at a smooth rate. We use pipettes pulled from Pasteur pipettes to the desired diameter, slightly larger than the size of the samples. Regular aspiration and adjusted pipette diameter ensure that the capsules are aligned and regularly separated in the tapered portion of the pipette. Once the full length of the thin portion of the pipette is filled with capsules (typically up to dozen in our case), they are gently expelled one by one in the wells of the *UniverSlide* (Fig. 2h–j). Movie 2 shows the complete sequence of capsules collection and chamber loading.

At this stage, the samples (here, capsules) are positioned in the wells. Deposition of the lid on top of the chamber with protruding PDMS seal allows to achieve permissive sealing, which does not prevent gas exchange but is leak-free and evaporation-free. It is worth noting that the design of the wells, especially by their depth (of at least 5 times the size of the samples) permits an easy manipulation and transport of the chamber from bench to incubator and microscope, back and forth, with no risk of mixing up the individualized samples.

### Compatibility of the chamber with different microscopy techniques

**Figure 3:**
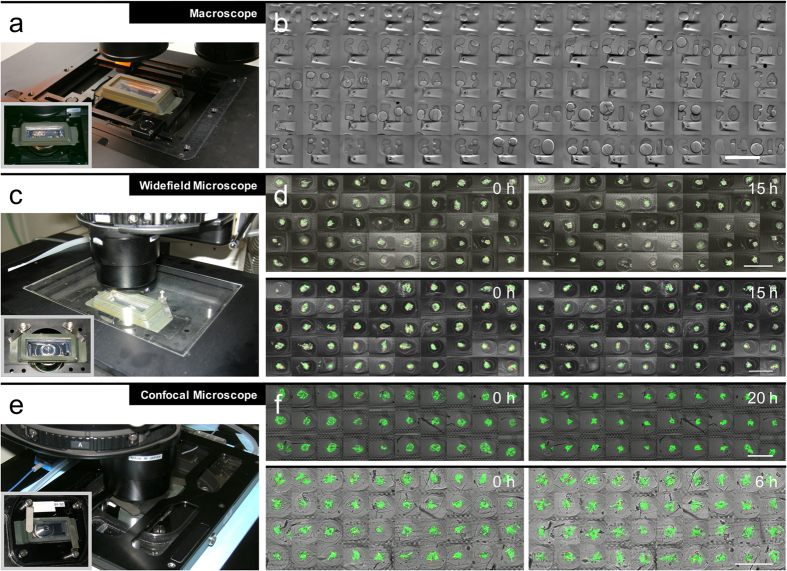
Imaging with different microscopes and modalities. (**a**) The chamber placed on the stage of a macroscope. (**b**) Tile of 70 fields of view acquired in bright field mode, comprising both the capsules in the wells, and the tag imprinted in the agarose gel. (**c**) The chamber placed in the stage of the widefield microscope covered by an insert for controlling gas and temperature. (**d**) Two overnight experiments (top and bottom panels) at the beginning (left) of the movie and at the end (right). (**e**) The chamber placed in the stage holder of the confocal microscope covered by the environment controlling insert. (**f**) Two overnight experiments (top and bottom panels) at the beginning (left) of the movie and at the end (right). All bars are 500 μm.

First, we imaged the capsules with a macroscope in an upright configuration (Fig. 3a). A motorized programmable stage combined with a stage holder equipped with sliding arms is especially well adapted to facilitate medium/high throughput low magnification imaging. Here we acquired up to 70 fields of view that encompass all the wells and the corresponding tags (Fig. 3b). Due to the tiny size of the letters there might be bubbles formed above, but despite of that, the tags are still visible. Second, we used a widefield-epifluorescence videomicroscope to perform overnight time-lapse acquisition (Fig. 3c). In this case, the stage, which accommodates regular microscopy slides, is also equipped with an insert to control the atmosphere (temperature and gas controlled: 37 °C, 5% C0_2_, from Life Imaging Services, Switzerland). In a series of two different experiments, we imaged 50 individual capsules overnight repeatedly (Fig. 3d). The axial stepping interval was 8 μm for both cases. The first dataset (Fig. 3d upper panels) was acquired with a time interval of 30 min and up to 80 μm while the second dataset (Fig. 3d lower panels) was acquired with a time interval of 2 hours and up to 150 μm. Third, we used a laser scanning microscope (‘confocal’, Fig. 3e) to reach a sufficient optical sectioning amenable for 3D visualization and accurate cell tracking. An insert for controlled environment (37 °C, 5% C0_2_, from Tokai Hit; Japan) was added, and we were able to image typically 30–40 capsules within 45 minutes (28 time-steps, over 20 hours) through 200 μm with z-sectioning of 5 μm. (Fig. 3f).

### Potentiality for refined 4D image analysis

**Figure 4:**
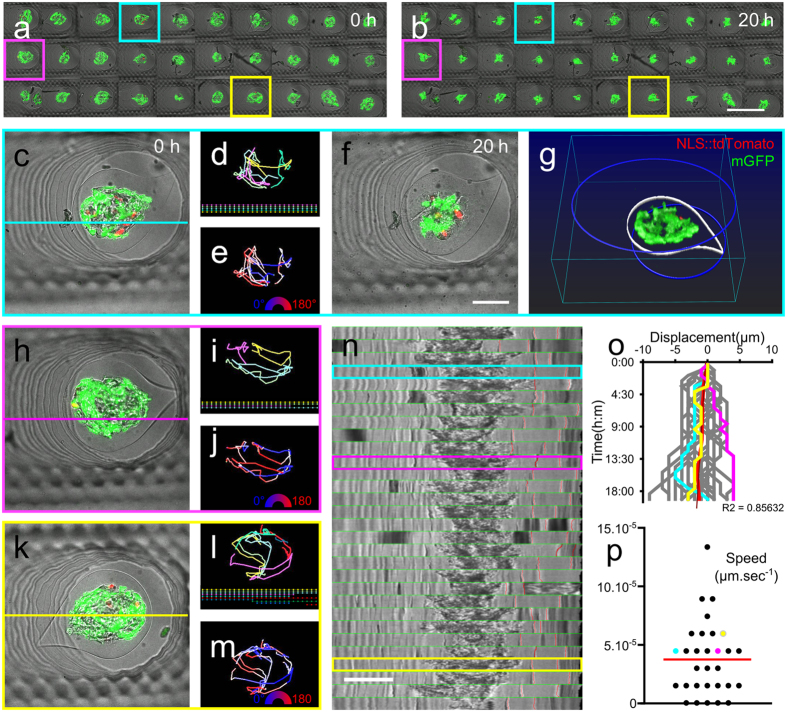
4D quantification of cells displacement within the capsules, a medium throughput analysis. (**a** and **b**) Tile images of the 30 fields of view imaged overnight with a 40-minute time-step at the beginning of the movie (**a**) and at the end (**b**). Field of view #4 (cyan) is exemplified in (**c**–**g**), #11 in h-j and #27 in k-m. For each capsule brightfield & fluorescence merge is shown at t0 (**c**,**h** and **k**) [and t + 20 h for #4 (**f**)]. Tracking of the tdTomato nuclei is represented according to individual cells (**d**,**i** and **l**) or traces are coloured according to directionality (**e**,**j** and **m**). (**g**) is a 3D rendering of the green and red cells, with the median plane of the capsule outlined (white), and the bottom and top border of the well delineated in blue. Analysis of the capsule motion inside the well is shown in (**n**–**p**). (**n**) Kymograph representation obtained from the bright field images in (**c**,**h** and **k**) by drawing a straight line horizontally in the middle of the field of view. (**o**) Plot of the displacement of the border of the capsule with respect to the border of the well (distance between thin red lines in n). (**p**) Scattered plot of the average speed of displacement computed from (**o**). Bar is 500 μm in (**b**) and 100 μm (**f**).

We focused on the dataset collected by confocal microscopy to perform image analysis. In Fig. 4, from all the fields of view collected with the same resolution of the whole *UniverSlide*, we selected three individual wells. For the sake of clarity, we provide composite images composed of a z-projection of the fluorescence channels overlapped with the median plane of the brightfield channel. In all cases the time course of cell displacements within the multicellular aggregate can be monitored (Fig. 4c,f,h and k; Movies 3, 5 and 7, right panels). We tracked the nuclei of the cell population labelled with NLS::tdTomato (Fig. 4d,e,i,j,l and m; Movies 3, 5 and 7, left panels). The trace for each cell (Fig. 4d,i and l) in each capsule shows a high mobility within the 3D microtissue. Typically, individual cells at the periphery of cellular aggregate may travel up to 300 μm (corresponding to half of the aggregate perimeter) within the duration of the acquisition (20 hours). Directionality analysis (colour rendering according to the angle of displacement in the image coordinates, Fig. 4e,j and m) is also shown to be feasible, which can be of great insight for deciphering how the two cell populations interact with each other. We also show that the spatial cell arrangement within the capsule can be monitored, as depicted in Fig. 4g, by the 3D rendering of the cellular content of the capsules (green and red cells) together with the contour of the capsule (white line) and the upper and lower borders of the wells (blues circles) [see also the related movies 4, 6 and 8]. This type of analysis was possible because the capsules exhibit minute or negligible movement inside the wells. Indeed, by monitoring the distance between the wall of the agarose well and the well of the capsule (Fig. 4n,o), we found that the displacement speed of the capsule was less than 0.2 μm.h^−1^ (Fig. 4p), corresponding to movements smaller than 5 μm within a day.

Here we have demonstrated the suitability of the chamber for imaging encapsulated spherical multicellular aggregates of a diameter between 100 and 250 μm in 3D and over long times. We have also shown that morphometric and biophysical analysis of cell dynamics during growth could be achieved. Although only demonstrated with alginate capsules filled with mammalian cell lines, we may readily anticipate this chamber to be equally adapted for ‘round’ embryos such as mammal embryos (mouse or rabbit for example) or ‘free-floating’ aquatic species embryos (sea-urchin, ascidian or amphioxus…).

### Adaptability to sample shape diversity: case example of the zebrafish larva

**Figure 5:**
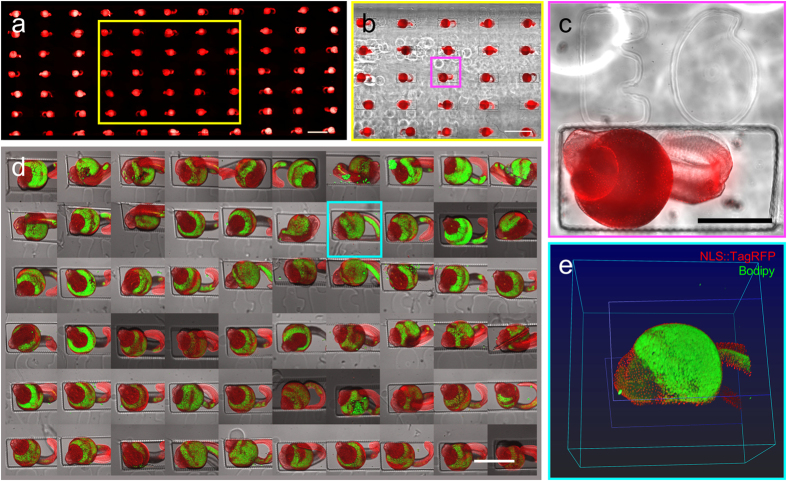
Transgenic zebrafish larva serial imaging in different conditions. (**a**–**c**) Widefield fluorescence and brightfield stitched images of large field of views. a. Stitching of a very large field of view acquired with a 4X objective and reconstructed by the acquisition software. (**b**) Central zone outlined in yellow in (**a**) is shown as an overlay of brightfield and fluorescence, this stitched view is acquired with a 10X objective. (**c**) Crop of the central embryo (magenta square in b) illustrates the resolution of imaging making discernible the single nuclei of each cell of the embryo. (**d**,**e**) Confocal imaging of the transgenic embryos counterstained with the lipid marker Bodipy. (**d**) Tile of the 60 fields of views acquired with confocal microscopy. Overlay of fluorescence projection and brightfield images. (**e**) 3D rendering of the two fluorescent channels and depiction of the bottom and top of the well (blue lines). All bars are 500 μm.

To extend further the capabilities of our imaging chamber, we also designed stamps dedicated to samples with different shapes and sizes. As a case example, we chose the zebrafish larva. Inspired by a previously proposed design^33^, we fabricated a stamp that generates parallelepipedic wells whose dimension match the size of the larva at the developmental stage of interest (0.7 × 1 × 1.5 μm, Supplementary Fig. S3f). We used a transgenic zebrafish line in ‘Casper’ background (transparent strain) that expresses ubiquitously TagRFP in the nuclei (NLS fusion). Larvae fixed at 30 hpf were imaged by widefield microscopy (Fig. 5a–c) or incubated in the lipid marker Bodipy 505/515 and imaged by confocal microscopy (Fig. 5d–e). Considering the size of the wells, we could accommodate 77 fishes (when the whole frame is completely filled, 11 columns and 7 rows) in a single chamber and thus image them in parallel. In Fig. 5a, using a 4X objective we performed a full scan of the chamber (corresponding to the 77 fishes) within 5:40 hours. Then a 10X objective was used to scan a rather smaller area (5 × 5 fishes, Fig. 5b) with a better resolution. Remarkably, this allowed us to distinguish single nuclei (Fig. 5c). Also note that the tag of the well is easily visible, which allows to record the developmental history of each individual fish, and then make the correspondence when further analyses are conducted.

## Discussion and Conclusion

In this work we present a device that facilitates imaging of 3D living objects irrespective of the shape of the sample or the type of microscopy technique. We provide the files for printing the different parts of the device using an inexpensive 3D stereolithography printer. We also describe protocols to achieve high-quality imaging in a medium/high throughput format. Our chamber allowed us to image up to hundreds of objects in 3D over time during 20 hours (which is not a technical upper limit) after a simple and rapid sample preparation. Although numerous sophisticated high resolution microscopy techniques are now available and widespread for imaging 3D cell cultures and model growing organisms, a practical and seemingly trivial limitation remains the sample manipulation and preparation for microscopy. We believe that the *UniverSlide* helps to overcome these difficulties. We analysed the datasets obtained at high resolution in 4D (3D + time) with dedicated tools and extracted the trajectories of a sub-population of individual cells inside multicellular spheroids. We collected data for about 30 capsules within the same run and performed, as an example, tracking for 3 capsules. Tracking was achieved without any problem, and so far, the only limitation seems the microscopy technique itself, e.g. penetration of light and scanning time. This limitation could be eventually overcame with the use of multi-photon microscopy with excitation infrared light to achieve a deeper penetration of light in thick samples and less phototoxicity due to out-of-focus excitation), or light sheet microscopy which consists in rapid scanning of a sheet of light (either real or digitally scanned) to greatly increase scanning speed. In the latter case a setup based on an upright collection and excitation optic path or with a 45° tilted optic axis could be an option (from either commercially available microscopes such as Leica DLS, 3i Lattice, or with sophisticated homemade microscopes^34^,^35^,^36^.

We have validated the usability of this chamber with two case examples: a spherical model organoid (mammalian cells encapsulated in alginate capsules) and an elongated vertebrate larva (zebrafish larva at 30 hpf). However, we expect this chamber to be suitable for any 3D biological sample (e.g. all sorts of embryos and organoids). The wide range of applications of this chamber makes it indeed potentially useful for a large number of laboratories. To disseminate this solution, all the design files are freely available, ready to be printed in the lab and assembled in a DIY way.

We have also shown that the *UniverSlide*, the design of which is based on a regular microscopy slide, is compatible with most of the commercially available stage holders, and even inserts for environment controlling systems. Moreover, the samples can be imaged either in an upright or in an inverted microscope, because both the bottom and the ceiling of the chamber are standard coverslips. The use of agarose gel for the support frame in which the wells are moulded is especially convenient because it is inert for most samples. Although not tested in the framework of this study, other transparent hydrogels such as Methyl Cellulose or Phytagel (Sigma) could a priori be used equally. Similar to chambers commercially available for 2D cell culture, the removable lid enables the experimentalist to alternate microscopy sessions with resting periods in the cell culture incubator and to facilitate medium exchange.

Each sample is also individually identified with a code assigned to each well, permitting the tracking of the imaged objects over further experiments (nucleic acid or protein sequencing, immunostainings…). This tag is visible even when the focus is made on the sample in the bottom of the well and not on the surface.

To conclude, we provide the experimentalist a convenient tool that is suited for a variety of biological applications ranging from cells to living organisms, in many contexts from cancer research to developmental biology. We believe that this tool overcomes the limitations of existing devices and will spur research in many areas of the life sciences.

## Material and Methods

### 3D printing and procedure for chamber parts production

#### Generalities

3D printing encompasses a large variety of techniques. The chamber we describe in this article was designed to be printed with a low cost Digital Light Processing (DLP) 3D printer.

The minimal specification to meet is a voxel resolution smaller than 125,000 μm^3^ (50 μm in each dimension).

The parts were designed and drawn with the Computer Assisted Drawing (CAD) software SolidWorks (Dassault System). The same program was used to generate the 3D renderings of the different parts (Fig. 1a, Supplemental Fig. S2a) as well as for generating all the STL format files, which is the universal file format input for most of the 3D printers.

We used the desktop format Micro Plus Hi-Re 3D printer (Envisiontech, CITY) that provides high resolution (Voxel: 25 μm in z, 30 μm in y and 40 μm in x). The selected resin (HTM140, Envisiontech) is specifically a high precision resin which allows to print protruding details as small as 150 μm or 6 pixels in diameter. In addition, this resin was developed to sustain high temperature (up to 140 °C), organic solvent (such as ethanol) and UV, which allows many sterilization processes. Nevertheless, repeated treatments with UV or high temperature such as autoclave result in accelerate aging of the printing material which became more brittle.

#### Printing tips

The stamp was printed with maximal z-resolution [(30.7 × 39 × 25) μm^3^ voxels] because the patterns printed in relief on the stamp determine the shape of the wells that is designed to accommodate precisely the samples. The other components, i.e. the slide holder and the lid do not require such an accuracy. They were printed with (30.7 × 39 × 50) μm^3^ voxels resolution, which allows to reduce the construction time by 2-fold. Build styles and ‘STL’ files are included (Supplemental ‘STL’ Files 1–9).

Printing time was further optimized i) by choosing the orientation of the pieces relative to the printer’s platform in order to decrease the number of z-layers to be printed, and ii) by parallelising the printing, i.e. filling the platform areas with the maximum of prints.

The stamp was printed with the wells imprint facing the bottom of the printing tray to ensure the high resolution printing of the wells, the tip of which is a flat surface with a circular section of 300 μm in a diameter. The two-part handle was designed to be printed on the same run. The total printing time is around 1:30 h.

To ensure the parallelism of the frame, the slide holder is printed with its longer axis tilted with a 20° angle, in order. Our design has some abrupt transitions in between layers if printed straight (e.g. in between the head of the slide and the centre which is mainly empty). Such abrupt transitions tend to bend the piece under the load during the printing. On our printer, four slides can be fitted on the printing platform and the total printing time is around 12:00 h.

The lid and the mould for the PDMS seal can be printed on the same run, which takes 8 to 10 hours depending on the orientation of the components.

#### PDMS seal

The PDMS seal is moulded from the 3D printed parts. To avoid tearing of the PDMS frame upon peeling the plastic mould, we had to reduce the affinity between PDMS and the crosslinked resin. Three possibilities work equally: 1/ Glycerol coating by dipping; 2/ hydrophobization using Trichloro(1 H,1 H,2 H, 2H-perfluorooctyl)silane (product #448931, Sigma Aldrich). To do so, the pieces were placed in the plasma cleaner for 5 to 10 minutes, transferred in a petri dish with a 40 μL drop of silane and allowed to dry for 1:00 h under a fume hood; 3/ use of a specific resin optimized for silicone moulding (E-Silicon, Envisiontech).

Liquid PDMS (Sylgard 184 Corning, Neyco, France) is carefully mixed (9/1, base/curing agent, w/w), degassed under vacuum and poured into the assembled mould on the bottom of a 100 mm petri dish. After curing for 3 h, the PDMS block is peeled and ready to use.

#### Cells production

Two human cell lines were cultured in their corresponding growing medium at 37 °C and in 5% CO_2_. The first cell line was infected by membrane-GFP lentivirus and the second cell line by nuclear-tdTomato. Both lentivectors were a gift from Connie Cepko (Addgene #22479 and #37347). Details for lentiviral production and titration were previously described^37^.

#### Cells encapsulation

Multicellular aggregates were formed by encapsulation and growth in alginate hollow spheres. We used equivalently the Cellular Capsules Technology^23^ or the method developed by X. He’s co-workers^38^ or the one developed by C. Kim *et al*.^39^. Briefly, for the Cellular Capsule Technology a sodium alginate liquid solution and the cell suspension are injected in a microfluidic device that generate a compound jet of liquid with a layer of liquid alginate around the cell suspension liquid core. Due to the Plateau–Rayleigh instability the jet breaks into droplets with the same compound configuration, i.e. the liquid sodium alginate solution around and the cell suspension in the core. These droplets are then collected in a calcium bath which crosslink almost instantaneously the alginate making a shell of alginate gel around a liquid core enclosing the cells in suspension. Addition of Matrigel allows to form a layer that coats the inner wall of the capsules. Since the gel is permeable to nutrients, cells are able to divide and fill up the capsule in appropriate culture conditions. All experimental details can be found in ref. 32.

#### Fish line and staining

We used larva from a transgenic zebrafish line [beta-actin promoter driving the expression of NLS::TagRFP fixed at 30 hpf (hours post fertilization) (Prim5, ref. 40) in 4% PFA (purchased from the AMAGEN platform)].

A batch of larva was soaked in 60 μM of Bodipy 505/515 solution (D3921, ThermoFisher) for an hour and rinsed with PBS.

#### Imaging

Macroconfocal imaging was conducted on a Nikon AZ-100 stand equipped with a C2 scan head with the 2X objective (NA 0.2, WD 45, AZ-Plan Fluor, Nikon). Videomicroscopy (brightfield and epifluorescence) images were acquired on a Nikon Ti Eclipse stand, collected with an Orca R2 camera (Hamamatsu, Japan) through the following set of objectives: 4X (NA 0.13, WD 17.2, CFI Plan Fluor, Nikon), 10X (NA 0.3, WD 16, CFI Plan Fluor, Nikon) and a 20X (NA 0.45, ELWD 6.9–8.2, CFI S Plan Fluor, Nikon). Confocal images and movies were made with a Nikon Eclipse Ti equipped with a C2Si scan head and a 20X objective (NA 0.75, WD 1, PlanApo VC, Nikon). All of these scopes are equipped with computer controlled motorized stages (Nikon and Prior) that permit repetitive and reproducible scanning of all the wells within the chambers. All microscopes are equipped with the Nikon NIS Elements package software.

#### Image analysis

The NIS software (Nikon) was used to export the acquired files into tiff format. Pre-processing, denoising, filtering and basic analysis were achieved with Fiji (https://fiji.sc/). 3D renderings were done with the USCF Chimera software (https://www.cgl.ucsf.edu/chimera/). Manual individual cell tracking was done with the standalone version of the MovIT software (http://bioemergences.eu/bioemergences/openworkflow-index.php and ref. 41) developed by the BioEmergences platform (https://bioemergences.eu/). Quantifications and graphs were made with either Excel (Microscoft) or Prism6 (GraphPad).

## Additional Information

**How to cite this article**: Alessandri, K. *et al*. All-in-one 3D printed microscopy chamber for multidimensional imaging, the *UniverSlide. Sci. Rep.* **7**, 42378; doi: 10.1038/srep42378 (2017).

**Publisher's note:** Springer Nature remains neutral with regard to jurisdictional claims in published maps and institutional affiliations.

## Supplementary Material

Supplementary Movie 1

Supplementary Movie 2

Supplementary Movie 3

Supplementary Movie 4

Supplementary Movie 5

Supplementary Movie 6

Supplementary Movie 7

Supplementary Movie 8

Supplementary Movie 9

Supplementary Information

STL File
