# Supplementary material for: All-in-one 3D printed microscopy chamber for multidimensional imaging, the UniverSlide
Source: Sci Rep. 2017 Feb 10;7:42378. doi: 10.1038/srep42378 (PMC5301227; doi:10.1038/srep42378)
Supplement: Supplementary Information [file srep42378-s10.docx]

All-in-one 3D printed microscopy chamber for multidimensional imaging, the *UniverSlide*

Kevin Alessandri^1, 2, 3, *^, Laetitia Andrique^3, 4, *^, Maxime Feyeux^3, 5, *^, Andreas Bikfalvi^3, 4^, Pierre Nassoy^1, 2, 3^& Gaëlle Recher^1, 2, 3, $^

1. LP2N, CNRS UMR 5298

2. Institut d’Optique Graduate School

3. Université de Bordeaux

4. LAMC, Inserm U1029

5. IMN, CNRS UMR 5293

*. Equal contribution

$. Corresponding author, [gaelle.recher@institutoptique.fr](mailto:gaelle.recher@institutoptique.fr)

# Supplemental information

### Supplemental Figure 1

**
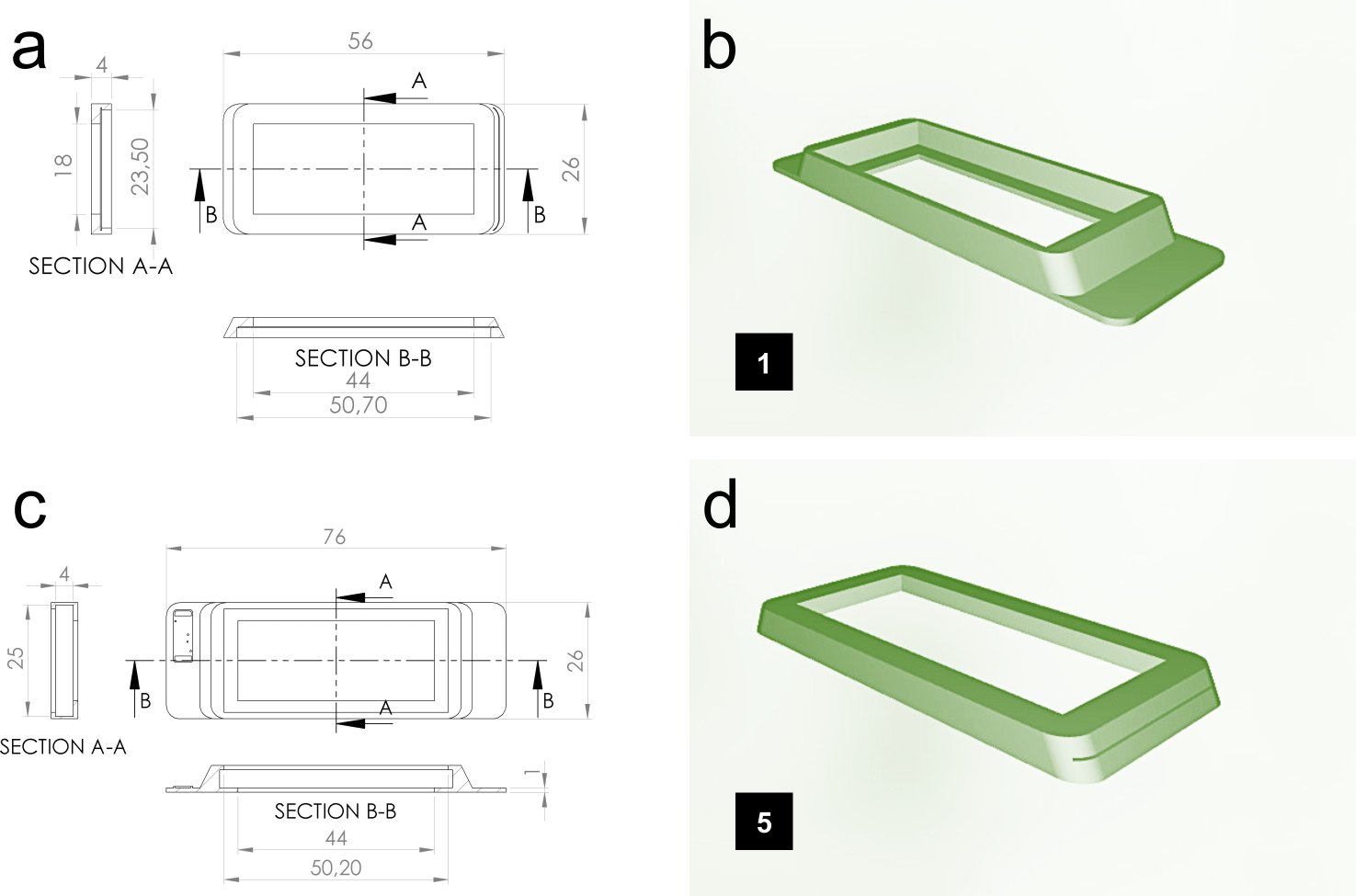
Supp Fig 1: Dimension and shape of the chamber and the lid.** a. Drawing of the chamber, the overall size (76*26 mm) corresponds to the standard size of a microscopy slide. b. 3D depiction of the corresponding STL file (found as a separate and downloadable file on the website of the journal). Number ‘1’ refers to Fig1. c. Drawing of the lid. d. 3D depiction of the corresponding STL file. Number ‘5’ refers to Fig1.

### Supplemental Figure 2

**
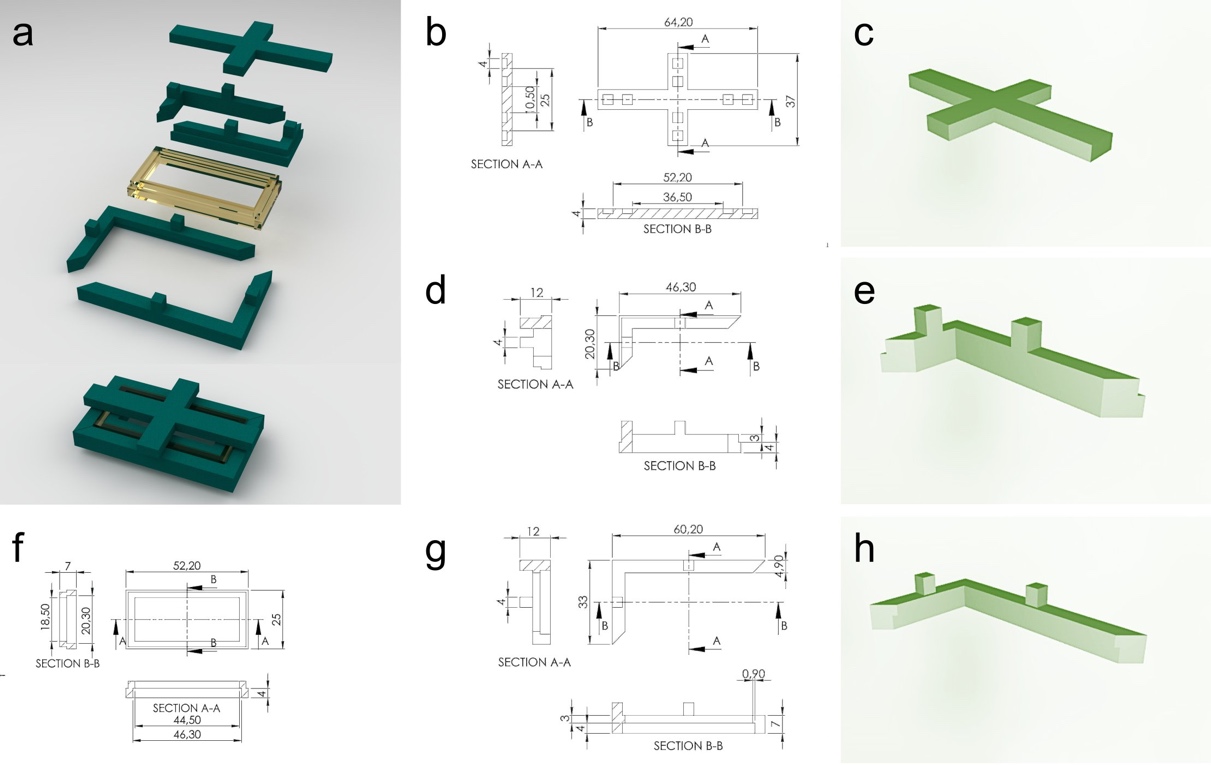
Supp Fig 2. Assembly, dimension and shape of the mould for making the PDMS seal.** a. Splitted and assembled 3D views of the mould assembly. An external and an internal frames are tight together with a cross that enable the overall structure to be set in place while pouring the PDMS within. b. Drawing of the cross. c. 3D depiction of the corresponding STL file. d. Drawing of the internal frame (the two halves are identical). e. 3D depiction of the corresponding STL file. f. Drawing of the resulting PDMS seal once solidified. g. Drawing of the external frame (the two halves are identical). h. 3D depiction of the corresponding STL file.

### Supplemental Figure 3

**
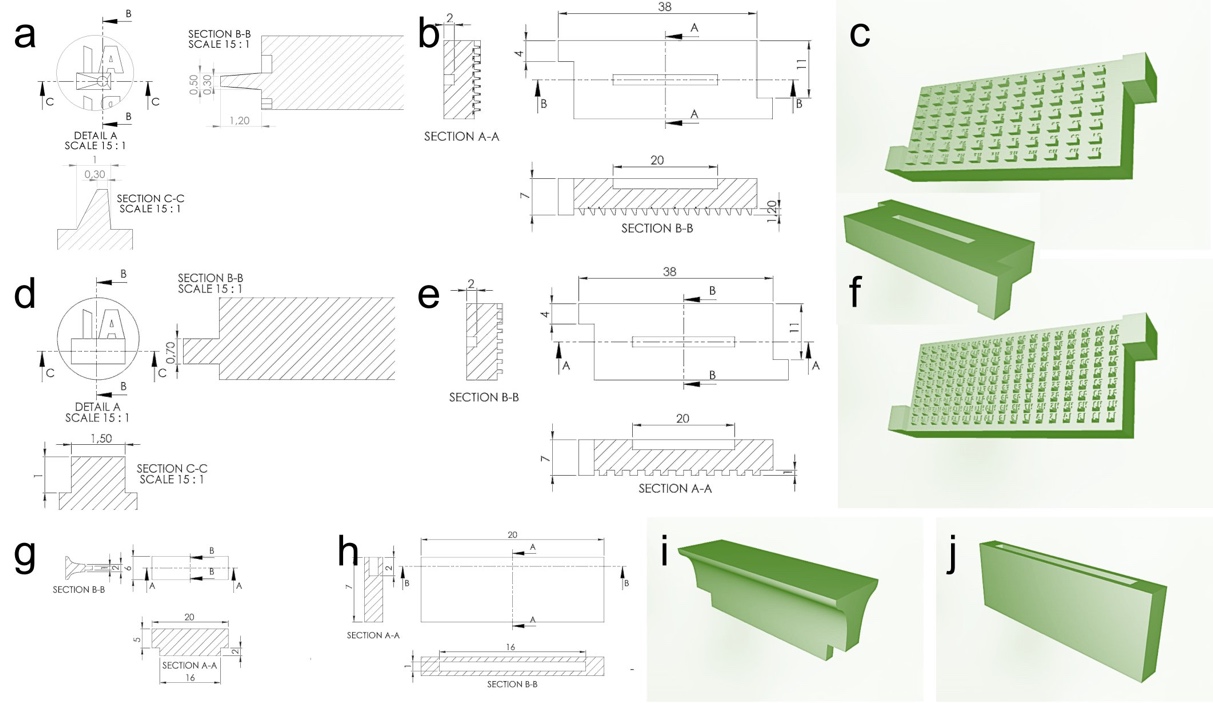
Supp Fig 3: Dimension and shape of the stamps.** a. Drawing of the stamp dedicated to small and spherical objects. b. Higher magnification of the well imprint. c. 3D depiction of the corresponding STL file. a. Drawing of the stamp dedicated to 30 hpf zebrafish larva. b. Higher magnification of the well imprint. c. 3D depiction of the corresponding STL file. g-h. Drawing of the two parts of the stamp handle. i-j. 3D depiction of the corresponding STL files.

### Supplemental Movie 1

**Supp Movie 1. Shape of the well.** 3D rendering of the shape of the well. Correspond to the screen capture shown in Fig 2d.

### Supplemental Movie 2

**Supp Movie 2. Loading the agarose pad with the capsules.** Movie showing the whole procedure for collecting the capsules within the petri dish and for deposing them in the agarose wells. The technique of collecting the objects with a mouth pipet and a pulled Pasteur glass pipet is derived from mouse embryology.

### Supplemental Movie 3

**Supp Movie 3. Cell dynamics in capsule #4.** On the left half, representation of the single cell trajectories. On the right half, overlay of the GFP and the tdTomato channels with the median plane of the brightfield channel. Related to Fig 4d, f.

### Supplemental Movie 4

**Supp Movie 4. 3D rendering of capsule #4.** 360° rotation of the 3D rendering of the cells (green and red), the capsule perimeter (white line) and the well top and bottom openings (blue lines). Related to Fig 4g.

### Supplemental Movie 5

**Supp Movie 5. Cell dynamics in capsule #11.** On the left half, representation of the single cell trajectories. On the right half, overlay of the GFP and the tdTomato channels with the median plane of the brightfield channel. Related to Fig 4h.

### Supplemental Movie 6

**Supp Movie6. 3D rendering of capsule #11.** 360° rotation of the 3D rendering of the cells (green and red), the capsule perimeter (white line) and the well top and bottom openings (blue lines). Related to Fig 4h.

### Supplemental Movie 7

**Supp Movie 7. Cell dynamics in capsule #27.** On the left half, representation of the single cell trajectories. On the right half, overlay of the GFP and the tdTomato channels with the median plane of the brightfield channel. Related to Fig 4k.

### Supplemental Movie 8

**Supp Movie 8. 3D rendering of capsule #27.** 360° rotation of the 3D rendering of the cells (green and red), the capsule perimeter (white line) and the well top and bottom openings (blue lines). Related to Fig 4k.

### Supplemental Movie 9

**Supp Movie 9. 3D rendering of a zebrafish larva.** 360° rotation of the 3D rendering of zebrafish larva (Bodipy in green and nuclei TagRFP in red), the well top and bottom openings (blue lines). Related to Fig 5e.

### Supplemental Files: STL files to print the different parts

1 to 9 for the different parts
